# ESFPNet: Efficient Stage-Wise Feature Pyramid on Mix Transformer for Deep Learning-Based Cancer Analysis in Endoscopic Video

**DOI:** 10.3390/jimaging10080191

**Published:** 2024-08-07

**Authors:** Qi Chang, Danish Ahmad, Jennifer Toth, Rebecca Bascom, William E. Higgins

**Affiliations:** 1 School of Electrical Engineering and Computer Science, Penn State University, University Park, PA 16802, USA; qxc62@psu.edu; 2 Penn State Milton S. Hershey Medical Center, Hershey, PA 17033, USA; danish.ahmad3141@gmail.com (D.A.); jtoth@pennstatehealth.psu.edu (J.T.); rbascom@pennstatehealth.psu.edu (R.B.)

**Keywords:** deep learning, endoscopic video analysis, autofluorescence bronchoscopy, colonoscopy, lung cancer, colorectal cancer, lesion analysis, semantic image segmentation, efficient stage-wise feature pyramid, mix transformer

## Abstract

For patients at risk of developing either lung cancer or colorectal cancer, the identification of suspect lesions in endoscopic video is an important procedure. The physician performs an endoscopic exam by navigating an endoscope through the organ of interest, be it the lungs or intestinal tract, and performs a visual inspection of the endoscopic video stream to identify lesions. Unfortunately, this entails a tedious, error-prone search over a lengthy video sequence. We propose a deep learning architecture that enables the real-time detection and segmentation of lesion regions from endoscopic video, with our experiments focused on autofluorescence bronchoscopy (AFB) for the lungs and colonoscopy for the intestinal tract. Our architecture, dubbed ESFPNet, draws on a pretrained Mix Transformer (MiT) encoder and a decoder structure that incorporates a new Efficient Stage-Wise Feature Pyramid (ESFP) to promote accurate lesion segmentation. In comparison to existing deep learning models, the ESFPNet model gave superior lesion segmentation performance for an AFB dataset. It also produced superior segmentation results for three widely used public colonoscopy databases and nearly the best results for two other public colonoscopy databases. In addition, the lightweight ESFPNet architecture requires fewer model parameters and less computation than other competing models, enabling the real-time analysis of input video frames. Overall, these studies point to the combined superior analysis performance and architectural efficiency of the ESFPNet for endoscopic video analysis. Lastly, additional experiments with the public colonoscopy databases demonstrate the learning ability and generalizability of ESFPNet, implying that the model could be effective for region segmentation in other domains.

## 1. Introduction

For patients at risk of developing either lung cancer or colorectal cancer, the identification of suspect cancerous lesions in endoscopic video is an important procedure. To perform an endoscopic exam, the physician navigates an endoscope through the organ system of interest and performs a visual inspection of the resulting video stream to identify suspect lesions. In particular, for the lungs, the physician performs an airway exam using a bronchoscope to identify suspect cancerous lesions developing along the airway walls [1,2]. For the colon, the physician draws on a colonoscope or wireless capsule endoscope to identify polypoid lesions (polyps) along the intestinal surface [3,4].

Unfortunately, while endoscopy has the advantage of being minimally invasive, the procedure forces the physician to perform a time-consuming, error-prone interactive search over a video stream consisting of thousands of frames. In addition, the large bulk of a typical exam video consists of normal findings and a considerable amount of distracting repetitive image data, making the task all the more challenging. A solution is to apply computer-based processing to the video.

In this paper, we propose a computer-based approach that enables the accurate real-time detection and segmentation of cancerous lesions in endoscopic video. While our development applies to endoscopy in general, our results and discussion focus on autofluorescence bronchoscopy (AFB) for the lungs and colonoscopy for the intestinal tract [1,2,3,4]. Regarding AFB, standard white-light bronchoscopy (WLB) initially was applied, with minimal success, to the detection airway (bronchial) lesions, but the later introduction of AFB showed a two- to six-fold increase in sensitivity to suspicious bronchial lesions compared to WLB [5,6]. Similarly, colonoscopy and wireless capsule endoscopy have been gaining acceptance recently for detecting colorectal cancer, with much ongoing effort for developing reliable methods for detecting colonic polyps [4,7].

We emphasize that the issues with the interactive video analysis highlighted above significantly hinder the accuracy and routine use of endoscopy for cancer detection. To appreciate this point, recent AFB studies have shown lesion detection sensitivities varying from 44% to 82%, amply pointing out the performance variations between physicians [2]. Regarding interaction time, an AFB study reported a mean exam inspection time of 15–20 min [8]. This is in contrast to the routine non-diagnostic WLB airway exam generally performed before all bronchoscopies, which takes on the order of 2 min. Similarly, for wireless capsule endoscopy, gastroenterologists reported spending 30–40 min to read the image data from one exam [4]. Automated computer-based methods, which have proven their value in other imaging applications, could greatly ease these limitations and help make endoscopy a more useful tool for early cancer detection.

Initial computer-based approaches for processing endoscopic video drew on traditional methods, consisting of image processing operations, hand-crafted image features, and rudimentary pattern recognition techniques [9,10,11,12,13,14], while, for AFB, a simple R/G ratio method based on the ratio of AFB’s red reflectance and green fluorescence signals has seen use, but with limited success, for bronchial lesion detection [15,16]. Unfortunately, these approaches have not proven to give robust lesion segmentations, are subject to excess false detections, and cannot process a video stream near real-time, thereby making them unsuitable for practical endoscopic examination.

More recently, for colonoscopy, deep learning approaches have shown promise for mitigating these issues [17,18,19,20]. As an example, Unet++ adds densely connected nested-decoder subnetworks to the Unet architecture for semantic medical image segmentation [21,22]. It also uses a deep supervision mechanism to allow for improved feature aggregation across different semantic scales. Although Unet++ can provide more accurate segmentations than Unet, the model’s dense connections demand extensive computation. As a more recent example, the CaraNet also utilizes deep supervision to enhance the use of aggregated features [19]. Yet, in contrast to the complex subnetworks of Unet++, the CaraNet includes the advantageous self-attention mechanism and draws on the context axial reverse-attention technique on a pretrained Res2Net backbone [23]. Hence, it enables faster processing (sans GPU usage) and better segmentation performance than Unet++ when tested over multiple public colonoscopy datasets. Nevertheless, the CaraNet’s self-attention mechanism is complex.

On another front, the SegFormer has shown much success for the general computer vision task of semantic image segmentation [24]. The SegFormer provides a simple and efficient layout utilizing the attention technique referred to as “Mix Transformer (MiT) encoders”. Expanding upon the SegFormer, the SSFormer architecture extracts and aggregates local and global step-wise features from pretrained MiT encoders to predict abnormal regions [20]. Tests with publicly available colonoscopy datasets again demonstrate the performance and generalizability of the SSFormer (and its use of the MiT encoders) over CaraNet and Unet++. Yet, the feature pyramid used by SSFormer could be made more efficient, thereby reducing the processing time and network complexity. Lastly, we point out that, to date, no deep learning methods has been devised for AFB lesion analysis.

We propose a deep learning architecture that enables the real-time detection and segmentation of lesion candidates from endoscopic video. Our architecture, dubbed ESFPNet, draws on a pretrained Mix Transformer (MiT) encoder as the backbone and a decoder structure that incorporates an efficient stage-wise feature pyramid (ESFP) to promote accurate lesion segmentation. Overall, in comparison to existing deep learning models, the ESFPNet model often facilitates faster computation and requires fewer network parameters while also often giving better segmentation and detection performance. Also, the ESFPNet has demonstrable learning ability and generalizability, implying that it could be effective for region segmentation in other domains. Experiments with AFB and colonoscopy databases assert these observations.

Section 2 details the ESFPNet model’s architecture and design considerations. Section 3 next presents two sets of experiments. The first set draws on a database consisting of videos from AFB airway exams of lung cancer patients collected at our university hospital. (We note that no publicly available AFB database exists, and we now make ours available to the public.) The second set of experiments uses multiple publicly available colonoscopy video databases. We also use these databases to assess the learning ability and generalizability of our model. Finally, Section 4 gives a discussion and thoughts on future work.

## 2. Methods

The basic input is an endoscopic true-color video frame I consisting of 720 × 720 pixels. The proposed deep learning architecture ESFPNet performs a real-time analysis on $\hat{I}$, a preprocessed version of I, to output a segmented frame M consisting of regions denoting candidate lesions. Section 2.1, Section 2.2 and Section 2.3 describe the proposed ESFPNet architecture, while Section 2.4 gives the implementation details.

### 2.1. Proposed Architecture

**Figure 1:**
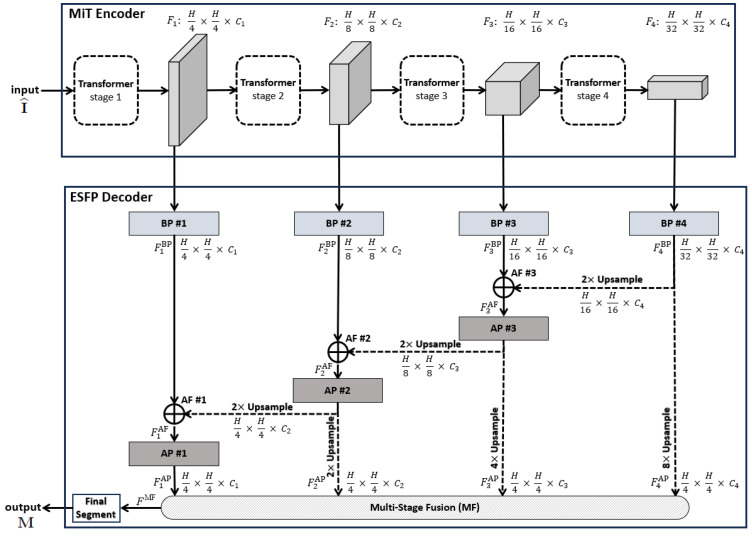
Top-level ESPFNet architecture. The input is an H×H video frame $\hat{I}$, where H=352 for our application, while the output is a 720×720 segmented frame M. The MiT encoder of Xie et al. serves as the network backbone [24], while the the Efficient Stage-Wise Feature Pyramid (ESFP) serves as the decoder. The four layers constituting the ESFP are (1) the basic prediction (BP) layer, given by blocks **BP #1** through **BP #4**; (2) the aggregating fusion (AF) layer, defined by **AF #1** through **AF #3**; (3) the aggregating prediction (AP) layer, given by **AP #1** through **AP #3**; and (4) the multi-stage fusion (MF) layer. The “Final Segment” block produces the final segmented video frame. Quantities such as $F_{1},F_{2},\ldots ,F^{\mathrm{MF}}$ denote the feature tensors produced by each network block, while quantities “$A\times A\times C_{i}$” specify the feature tensor dimensions, e.g., the dimensions of $F_{1}$ are $\frac{H}{4}\times \frac{H}{4}\times C_{1}$.

**Figure 2:**
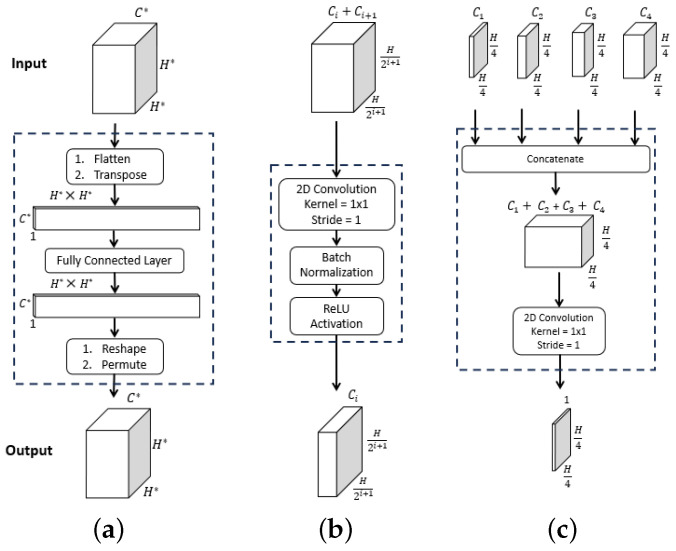
ESFPNet decoder modules. Inputs and outputs are given by the top and bottom feature tensor blocks, with dimensions as indicated. For the ConvModule(·), dimension $C_{i}$ arises from stage output $F_{i}$ of the MiT encoder. (**a**) Linear_Layer(·). (**b**) ConvModule(·). (**c**) Multistage_Fusion(·).

Our proposed ESFPNet network model (Figure 1) is at the heart of our approach, with Figure 2, Table 1, and the remainder of this section giving full details. The aim of the model is to achieve high lesion detection/segmentation accuracy and high computational throughput, while also reducing the number of parameters to tune for a given endoscopy application.

Note that state-of-the-art deep learning architectures generally require a large amount of data for adequate training and testing (the so-called “data hunger” problem) [25]. This is because the large number of parameters constituting many network architectures requires considerable tuning when training from scratch. As Section 3 later shows, the ESFPNet uses a pretrained encoder along with a lightweight decoder, which enables successful domain adaptation to new inputs despite the amount of available training data.

A pretrained 4-stage Mix Transformer (MiT) encoder serves as the ESFPNet encoder, while the proposed lightweight Efficient Stage-Wise Feature Pyramid (ESFP) serves as the architecture’s decoder to generate segmented lesion predictions. In this way, we leverage and focus on the feature extraction capabilities of MiT encoders, while only needing to additionally fine-tune the smaller number of parameters in the feature pyramid to an application’s dataset.

Each 720 × 720 video frame I undergoes the following preprocessing. First, it is center-cropped to a 704 × 704 array and downsampled to an H×H array (H= 352) for computational efficiency. These dimensions comply with the requirement of the encoder’s final stage that the output feature tensor must be a factor of H/32. Specifically, the 704 × 704 cropped image equals 2 × 352, and 352 = 11 × 32. These dimensions also strive to maintain the dimensions of the entire original bronchoscopic video frame. The next preprocessing operation entails applying intensity normalization to I to give $\hat{I}$. This commonly applied operation, used by the ImageNet and many other architectures, helps to improve convergence and training stability, while maintaining the relationships between minimum and maximum feature values [26]. Notably, both the CaraNet and SSFormer, used as example models in our later Section 3, also follow this approach, as their pretrained encoders are based on the ImageNet. Thus, $\hat{I}$ serves as the network input, while the output is a segmented 720 × 720 binary-valued video frame M. Section 2.2 and Section 2.3 give further detail on the ESFPNet encoder and decoder.

### 2.2. Backbone MiT Encoder

CNN-based encoders as utilized by the Unet, Res2Net, and SegNet architectures have enjoyed much success for image segmentation (CNN = convolutional neural network) [21,23,27]. A CNN-based encoder, motivated by the idea that every image pixel depends on its neighboring pixels, uses filters on an image patch to extract relevant local features. Yet, if a processing model utilized all image data (thereby taking a global view) instead of only the patches considered by the filters, then processing performance would be expected to improve. This concept helps explain why the so-called vision transformers (ViTs) work better than most CNN models for many feature-based computer vision tasks [28].

For the ESFPNet backbone, we draw on the Mix Transformer (MiT) encoder, summarized in Figure 1. The MiT encoder takes advantage of the idea of the ViT network by using four overlapping path-merging modules and self-attention prediction in four stages [24]. These stages not only furnish high-resolution coarse features but also provide low-resolution fine-grained features.

Using transformers as encoders, however, has a known limitation. The self-attention layers used by transformers lack locality inductive bias (i.e., the notion that image pixels are locally correlated and that their correlation maps are translation invariant) and require costly training on large datasets [28,29]. To alleviate this challenge, one can exploit the widely used concept of transfer learning to adapt to different problem domains. For our ESPFNet architecture, we perform this by integrating MiT encoders pretrained on the large ImageNet database [26], using the identical encoders employed by the SegFormer model of Xie et al. [24]. Subsequently, we propose three different versions of the ESFPNet architecture, based on the different MiT encoder scales available: (1) ESFPNet-T (tiny model); (2) ESFPNet-S (standard model); and (3) ESFPNet-L (large model). These encoders draw on the MiT-B0, -B2, and -B4 encoders, respectively, as detailed in the ArXiv version of the paper by Xie et al. [24]. Specifically, MiT-B0 uses $C_{1}=32$, $C_{2}=64$, $C_{3}=160$, and $C_{4}=256$ for each stage, respectively, while MiT-B2 and MiT-B4 use $C_{1}=64$, $C_{2}=128$, $C_{3}=320$, and $C_{4}=512$. Subsequently, we then train with a dataset for a specific endoscopy application (bronchoscopy or colonoscopy) in conjunction with our ESFP decoder. This proves to facilitate a performance level that often exceeds that of state-of-the-art CNN models.

**Figure 3:**
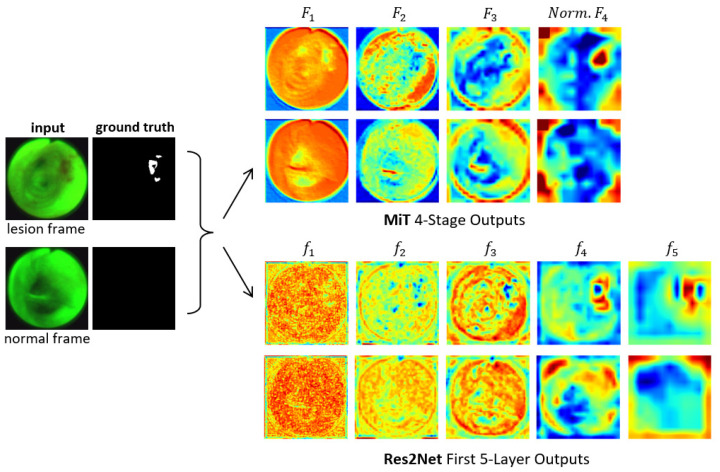
Attention heat maps of feature flow through two encoders for two example AFB video frames. Frames #1722 and #4182 representing lesion and normal frames, respectively, from patient case 21405-184 are considered. The “ground truth” frames denote ground truth segmented images $M^{\mathrm{GT}}$. The top two output rows are for the MiT-B2 encoder (Figure 1). For each feature tensor $F_{i}$, the corresponding heat map’s value at a given location equals the average of the computed $C_{i}$ features. For better visualization, the heat maps display the quantity 255−F; also, $F_{4}$’s output is normalized. The bottom two output rows are for the Res2Net encoder as used, for example, by CaraNet [19].

Figure 3 clearly depicts the functional superiority of the MiT encoder in comparison to the Res2Net encoder [23]. For the example AFB video frames, the MiT encoder effectively concentrates the model’s attention on critical details and generates valuable features right from the initial stage, as exemplified by the $F_{1}$ through $F_{3}$ outputs. The Res2Net encoder, on the other hand, remains focused on local patch information in the first 2–3 stages, resulting in more random appearing (and less useful) features. Not until stages 4–5 does the Res2Net encoder finally produce more apparent, but still vague, lesion information, due to the enhancement from its axial reverse attention blocks. In contrast, the MiT encoder’s final stage clearly indicates the lesion’s position. As a consequence, the MiT encoder offers more beneficial low- and high-level features for use in subsequent calculations. Section 3 later demonstrates that these features boost segmentation performance.

### 2.3. Efficient Stage-Wise Feature Pyramid (ESFP) Decoder

The prediction results of the decoder rely on multi-level features from the encoder, where local low-level features are extracted from the shallow parts of the encoder, while global high-level features are extracted from the deeper parts. Previous research has shown that the local features computed by the transformer’s shallow part significantly affect the model’s performance [30]. The existing SegFormer model, however, equally concatenates these multi-level features to predict segmentation results. Hence, it lacks the ability to sufficiently and selectively use the local features [24]. To address this issue, the SSFormer architecture includes an aggregating feature pyramid architecture that first uses two convolutional layers to preprocess feature outputs from each MiT stage. It then fuses any two features in reverse order from deep to shallow until final prediction [20]. In this way, local features gradually guide the model’s attention to critical regions.

Note, however, that global features typically contribute more to overall segmentation performance than local features, being especially useful for flagging regions of interest (e.g., lesions). Although the SSFormer enhances the contribution of local features, its usage of global features is weaker. In particular, its third stage is the one that derives features to flag lesion locations, while the feature information from its final fourth stage do not seem beneficial to the final outputs. Furthermore, its usage of the local emphasis layer is inefficient in that it wastes floating-point operations after the direct upsampling of features used for the later aggregating prediction layers.

Inspired by the structure of the lightweight channel-wise feature pyramid network (CFPNET) [31], we propose the efficient stage-wise feature pyramid (ESFP) to exploit multi-stage features. As Figure 1 summarizes, the ESFP decoder takes the four stage outputs of the MiT Encoder as inputs and consists of four successive linear layers:

1. Basic prediction (BP) layer;
2. Aggregating fusion (AF) layer;
3. Aggregating prediction (AP) layer;
4. Multi-stage fusion (MF) layer.

Referring to Figure 1, the data flow through the four decoder layers for input video frame $\hat{I}$ proceeds as follows. First, ESFP passes the four MiT encoder stage outputs $F_{i},\;i=1,2,3,4,$ through the basic prediction (BP) layer to produce the output feature tensors:

(1) $$F_{i}^{\mathrm{BP}}=\mathrm{Linear}\_\mathrm{Layer}\left(F_{i}\right)\;,\;i=1,2,3,4.$$

The aggregating fusion (AF) layer then linearly fuses these preprocessed features from global to local via

(2) $$F_{i}^{\mathrm{AF}}=\left\{\begin{array}{cc} \mathrm{ConvModule}\left(\mathrm{Concat}(F_{i}^{\mathrm{BP}},U_{2}\left(F_{i+1}^{\mathrm{AP}}\right))\right)\;, & i=1,2\\ \mathrm{ConvModule}\left(\mathrm{Concat}(F_{3}^{\mathrm{BP}},U_{2}\left(F_{4}^{\mathrm{BP}}\right))\right)\;, & i=3 \end{array}\right.$$

where the standard operations $\mathrm{Concat}(F_{1},F_{2})$ concatenate feature tensors $F_{1}$ and $F_{2}$, and $U_{i}\left(F\right)$ upsamples feature tensor *F* by a factor of *i* in both width and height. Next, the fused features pass onto the aggregating prediction (AP) layer to give outputs

(3) $$F_{i}^{\mathrm{AP}}=\left\{\begin{array}{cc} \mathrm{Linear}\_\mathrm{Layer}(F_{i}^{\mathrm{AF}})\;, & i=1,2,3\\ F_{4}^{\mathrm{BP}}\;, & i=4 \end{array}\right.$$

where (3) trivially defines $F_{4}^{\mathrm{AP}}$ for clarity. The intermediate aggregated features from all stages are then concatenated and fed into the final multi-stage fusion (MF) layer:

(4) $$F^{\mathrm{MF}}=\mathrm{Multistage}\_\mathrm{Fusion}\left(\mathrm{Concat}\left(F_{1}^{\mathrm{AP}},U_{2}\left(F_{2}^{\mathrm{AP}}\right),U_{4}\left(F_{3}^{\mathrm{AP}}\right),U_{8}\left(F_{4}^{\mathrm{AP}}\right)\right)\right)$$

Bold quantities Linear_Layer(·), ConvModule(·) and Multistage_Fusion(·) in Equations (1)–(4) signify the network components pictured in Figure 2, while Table 1 provides specific details for all blocks within a layer. As a final operation, $F^{\mathrm{MF}}$ passes through the **Final Segment** block consisting of the following operations:

1. Sigmoid activation;
2. Threshold >0.5;
3. 4× upsample;
4. Zero padding.

to produce a final binary-valued 720 × 720 segmented image M.

**Figure 4:**
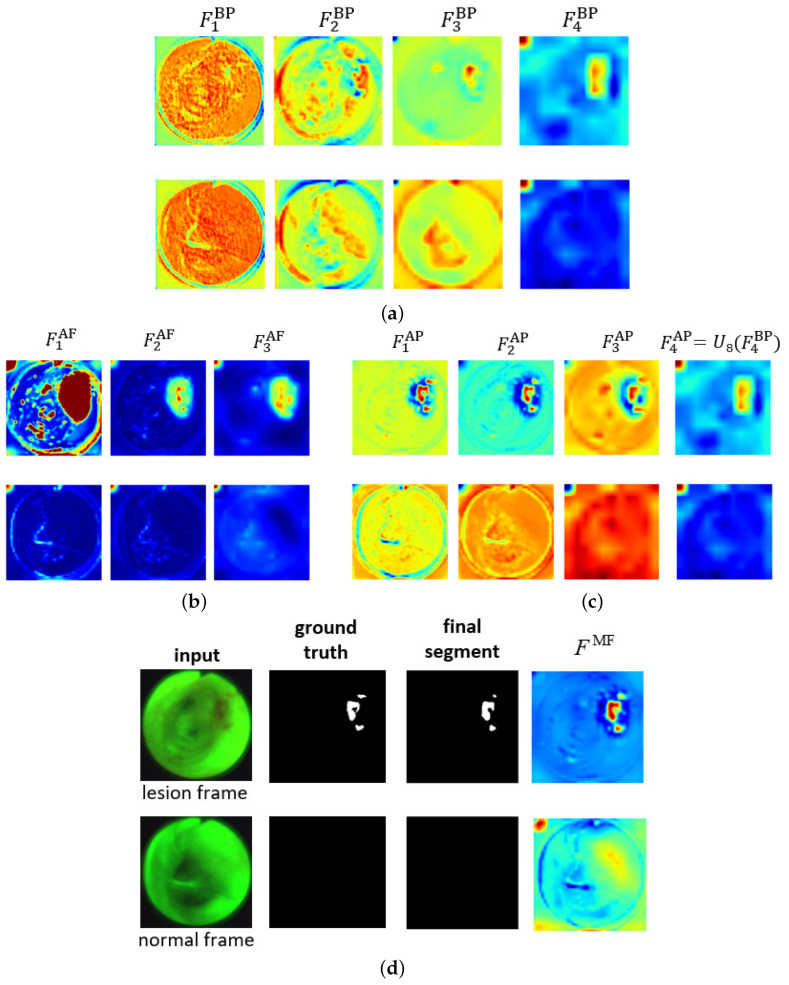
Attention heat maps of feature flow through the ESFPNet decoder for the two AFB frames of Figure 3. The top and bottom rows for each figure part correspond to lesion frame #1722 and normal frame #4182, respectively. The top right part of Figure 3 gives the decoder inputs. The first three AP heat maps display the quantity 255−F for better visualization. (**a**) Basic prediction (BP) outputs. (**b**) Aggregating fusion (AF) outputs. (**c**) Aggregating prediction (AP) outputs. (**d**) Ground truth and final segmentation outputs.

Figure 4 shows the ESFP decoder outputs for all stages for the two AFB input images considered in Figure 3. The figure clearly shows that the step-wise linear fusion of features from the various stages generates prediction heat maps that progressively incorporate local details and increasingly delineate lesion areas with greater accuracy. By doing so, the method effectively narrows the information gap between the high- and low-level features that are fused. In addition, by linearly fusing features from all stages, the lesion and normal regions are more clearly distinguished in $F^{\mathrm{MF}}$ as opposed to $F_{1}^{\mathrm{AP}}$, resulting in a strong lesion prediction. This occurs because the high-level features from $F_{4}^{\mathrm{AP}}$, derived directly from $F_{4}^{\mathrm{BP}}$, play a more significant role in the process. This results in low values for the normal region and high values for the lesion region, thereby highlighting the benefit of fusing features from all levels. As a result, the ESFPNet enables better performance than other models for single-frame lesion detection as shown later in Section 3.

### 2.4. Implementation Details

All network models were implemented in Python using the PyTorch framework. A Dell Precision 7920 Windows-10 PC, driven by an Intel Xeon Gold 6230 CPU @2.10 GHz with 26 cores and 64 GB RAM memory and equipped with an Nvidia GeForce RTX 3090 GPU with 24 GB GPU memory, was used for the majority of the training, validation, and testing. Due to the varying model sizes, which increases GPU memory demands, we utilized the Nvidia TESLA A100 GPU to train the ESFPNet. We also integrated C++ (version 14) versions of our models for use in our laboratory’s custom image-guided bronchoscopy software system [32,33]. Section 3 gives complete detail on all training, validation, and testing of the models.

## 3. Results

Section 3.1 and Section 3.2 compare the performance of the ESFPNet to other existing approaches for single-frame lesion analysis in endoscopic video over two domains: (1) autofluorescence bronchoscopy; and (2) colonoscopy. Finally, Section 3.3 discusses computational considerations.

### 3.1. Autofluorescence Bronchoscopy

We collected and recorded a series of AFB airway exams for 20 lung cancer patients scheduled for diagnostic bronchoscopy at our University hospital. All participants provided informed consent in accordance with an IRB protocol approved by our university’s Office of Research Protections. All exams were performed in the operating room under standard clinical conditions. The physician started an exam in the trachea and then scanned the following major airways: right main bronchus (RMB), right upper lobe bronchus (RUL), right lower lobe bronchus (RLL), left main bronchus (LMB), left upper lobe bronchus (LUL), and left lower lobe bronchus (LLL). Olympus BF-P60 bronchoscopes and the Onco-LIFE autofluorescence light source and video camera were used for all airway exams. The 20 recorded videos were collected at a rate of 30 frames/s and consisted of 66,627 total video frames. The recorded video sequences ranged in duration from 1 min 3 s to 3 min 34 s (median, 1 min 45 s), with video frame counts ranging between 1890 and 6426 frames (median, 3149 frames).

**Figure 5:**
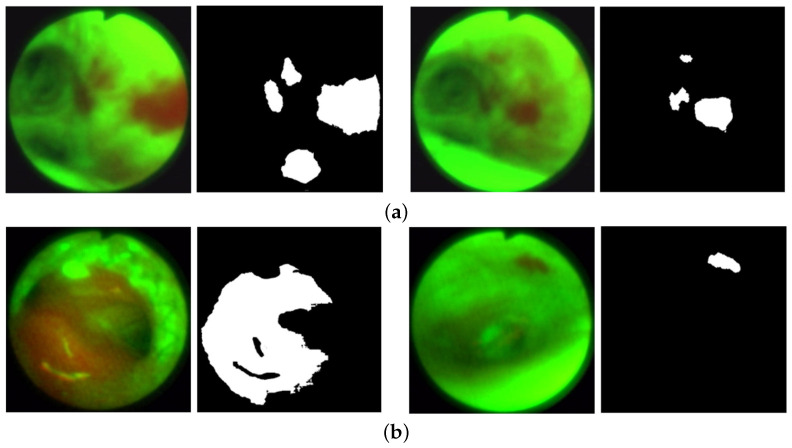
AFB lesion examples from the training and validation datasets. Each frame pair gives the original frame (left) and ground truth lesion segmentation (right). (**a**) Training dataset examples: case 21405-192, frames 1428 (left) and 1444 (right). (**b**) Validation dataset examples: case 21405-171, frames 0762 (left) and 5878 (right).

To perform the experiments, we created a 685-frame AFB dataset. Within the 20-case dataset, we selected 208 frames depicting clear ground truth bronchial lesions, where our selection strove to capture variations in airway location, lesion size, and viewing angles. Figure 5 gives sample lesion frames in the training and validation datasets. In addition, we incorporated 477 frames depicting normal conditions, chosen to represent a variety of airway locations and camera angles.

We point out that researchers have made a special point to note that segmentation methods not trained with any normal images often generate false positives on normal images; to solve this problem, a separate classification network may be used to classify frames as normal or abnormal [34,35]. For our application, by training with normal frames in the dataset, we provide added immunity to false positives and improve detection precision, without affecting the recall and mean Dice metrics derived for the validation dataset during training (all metrics are discussed further below). We observe that by doing so, all attention-based networks, such as CaraNet, SSFormer, and our proposed ESFPNet, do not erroneously detect lesions in normal images when the model converges. Lastly, the dataset includes more normal frames than lesion frames because such frames are far more common in a typical endoscopic exam.

An expert observer picked all lesion frames using the standard OpenCV CVAT annotation tool and defined segmentations through the MATLAB image labeler app [36,37]. Two to four hours were spent analyzing each video, with the inspection being time dependent on the video length and number of lesions. Up to three passes were made for each video to confirm frame choices, with two other experienced observers helping to corroborate decisions. We did not produce inter- or intra-observer agreement results to measure observer variations (Our anonymized dataset is available to the public on our laboratory’s web site under “Links/Public Databases” at Ref. [38]).

Per Table 2, the 685-frame AFB dataset was split into training, validation, and testing subsets using approximately a 50%, 25%, and 25% split, respectively. To avoid leakage between the data subsets, every lesion and normal frame from a given case was placed in the same subset to guarantee independence between the training, validation, and testing phases. Thus, because of this constraint, our actual splits into training, validation, and testing subsets were 47%, 28%, and 25%, respectively, as shown in Table 2. Lastly, the overall lesion regions roughly varied in size from 800 to 290,000 pixels within a video frame’s circular scan region made up of π·352·352 (≈390,000) pixels.

Over the complete 208-frame lesion dataset, a total of 128 distinct lesions were identified during ground-truth construction. Because a particular lesion is generally visible across multiple consecutive frames in a video sequence, considerable similarity will, of course, exist between adjacent, or nearly adjacent, video frames depicting a lesion. To eliminate the impact of frame correlation in the AFB dataset, 61 of the 128 distinct lesions were only represented by one frame in the dataset. For the remaining 67 lesions, we included one or more additional frames for a given lesion only if the added frames showed dramatic differences in size, viewing angle, or illumination. Because our focus is on single-frame detection, the lesion regions appearing in these added frames were all designated as distinct lesions in the dataset. Overall, the 208-frame lesion dataset depicts 311 regions representing lesions, with some frames depicting 1 or more lesion regions.

Note that our strategy for selecting multiple frames for a particular lesion is similar to that employed by other endoscopic imaging researchers. For example, with respect to the public colonoscopy datasets used in the next section, the CVC-Clinic colon database often depicts a particular polypoid lesion over six or more frames from a video, with each frame offering a distinct look [39]. Also, Urban et al. sampled every fourth video frame depicting a polyp for their dataset [40].

We compared the Unet++, SSFormer-S, SSFormer-L, CaraNet, and three ESFPNet models [19,20,22], along with traditional image-processing methods based on the simple R/G ratio and a machine-learning approach using a support vector machine (SVM) [12,16]. Chang et al. give details for the R/G ratio and SVM (only #1) methods used here [12]. Note that the UNet++ model had no pretrained components [22], while the CaraNet drew on a pretrained Res2Net encoder (see Figure 3) [19]. Finally, the SSFormer-S and SSFormer-L models used the same pretrained MiT-B2 and -B4 encoders, respectively, as those used by the ESFPNeT-S and ESFPNeT-L models.

All network models for the Unet++, CaraNet, SSFormer-S, SSFormer-L, and ESFPNet-T, ESFPNet-S, ESFPNet-L architectures were trained under identical conditions. We employed the Adam optimizer with learning rate = 0.0001, $\beta_{1}=0.9$, and $\beta_{2}=0.999$, similar to other recent endoscopic video studies conducted for the PraNet and CaraNet [19,41]. A network was trained for 200 epochs with batch size = 16, and image size = 352 × 352. To account for the imbalance in the number of normal and lesion frames, sampling weights for normal and lesion frames were set to 1.43 and 4.95, respectively, using the PyTorch function WeightedRandomSampler to ensure an equal number of normal and lesion frames (i.e., 8) in each training batch. We used the same loss

(5) $$L=L_{\mathrm{IoU}}^{w}+L_{\mathrm{BCE}}^{w}$$

function used by Wei et al. and Lou et al., where $L_{\mathrm{IoU}}^{w}$ and $L_{\mathrm{BCE}}^{w}$ are the weighted global intersection over union (IoU) loss and weighted local pixel-wise binary cross-entropy (BCE) loss, respectively [19,42]. The training process drew upon the training and validation datasets. During each training epoch, data augmentation techniques were applied to increase and diversify the training dataset. In particular, we employed randomized geometric transformations (rotation and flipping) and color jittering (image brightness and contrast changes), using methods built into PyTorch. Data augmentation, which helps reduce overfitting and improve network robustness, has been a standard procedure used for endoscopic video analysis, where large datasets are generally hard to compile [43]. Notably, all of the top teams, in a recent gastroenterology challenge, employed data augmentation [44].

To measure segmentation accuracy, we computed the mean Dice and mean IoU metrics:

(6) $$\mathrm{Dice}\left(A,B\right)=\frac{2|A\cap B|}{\left|A|+|B\right|}\;\;\;\mathrm{and}\;\;\;\mathrm{IoU}\left(A,B\right)=\frac{\left|A\cap B\right|}{\left|A\cup B\right|},$$

where *A* and *B* equal the segmented lesion and ground truth lesion, respectively, and |A| is defined as the area of *A*. All metrics were computed using tools along with PraNet [41].

As an additional goal, we also assessed lesion detection performance for the AFB dataset. We point out in passing that colonoscopy researchers have universally limited their focus to pixel-based region segmentation and have not considered region detection [19,20,22,45]. For our studies, any segmented region that overlaps a ground truth lesion was designated as a true positive (TP). A false positive (FP) corresponded to a segmented region, whether it be on a lesion or normal test frame, that did not overlap a ground truth lesion segmentation. Lastly, a false negative (FN) corresponded to a ground truth lesion not identified by a method. Given these definitions, we also used the following standard metrics to measure detection performance:

(7) $$\mathrm{recall}=\frac{\mathrm{TP}}{\mathrm{TP}+\mathrm{FN}}\;\;\;\mathrm{and}\;\;\;\mathrm{precision}=\frac{\mathrm{TP}}{\mathrm{TP}+\mathrm{FP}}\;,$$

where recall, or sensitivity, denotes the percentage of ground truth lesions detected, while precision, or positive predictive value, measures the percentage of segmented regions corresponding to correctly detected lesions.

**Figure 6:**
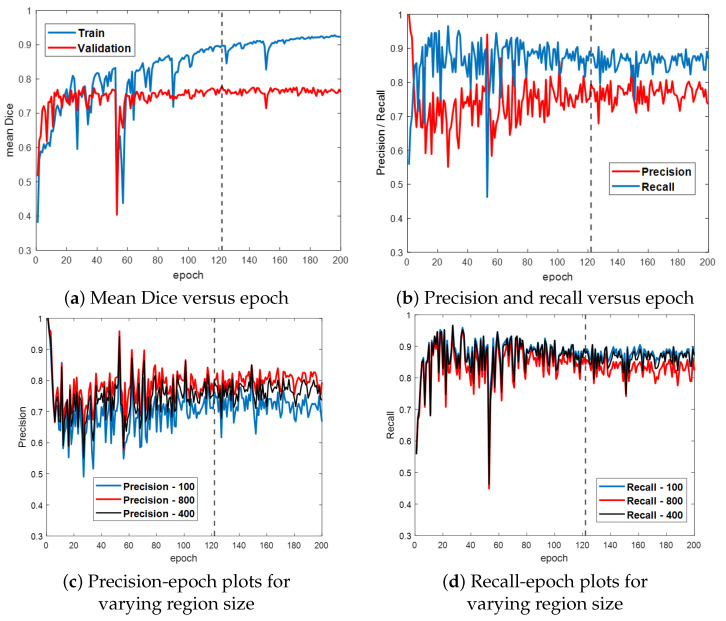
Training and validation results for ESFPNet-S. Part (**a**) plots the mean Dice index versus epoch for the training and validation data. Part (**b**) illustrates precision and recall performance versus epoch for the validation data. Parts (**c**,**d**) illustrate the impact of varying the size of a significant region on precision and recall performance. The dashed line indicates where our best model was selected at this epoch based on its performance on the validation data.

Figure 6 first gives the training and validation results for the ESFPNet-S model. Both the segmentation accuracy and detection performance (Figure 6a,b, respectively) steadily improve until leveling off around epoch 120, with little indication of overfitting. Based on these results, we froze model parameters at epoch 122. (Other models were similarly frozen by optimizing the mean Dice measure over the validation dataset.) Lastly, Figure 6c,d depict the impact of the significant region size parameter on detection performance. As this parameter varies from 100 (smaller regions retained), 400 (default value for later tests), and 800 pixels (stricter limit), the precision and recall performance results vary over a 5–10% range.

**Figure 7:**
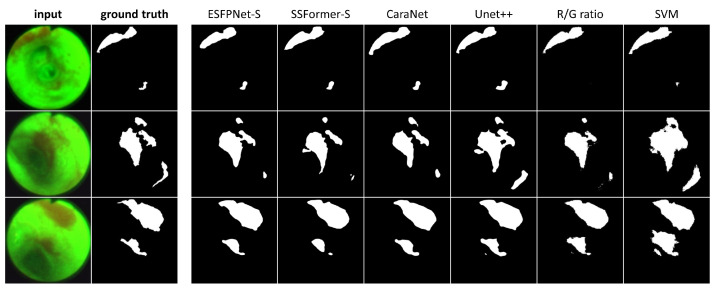
AFB Segmentation results. Each row corresponds to the following example AFB video frame: top row, case 21405-195, frame #1627; middle row, case 21405-184, frame #2549; bottom row, case 21405-184, frame #2580. The first two columns in each row depict the original video frames and ground truth segmentations, while columns 3 through 8 show segmentations derived by the various models ordered from the highest to lowest mean Dice index.

Table 3 next gives results for the AFB test set, while Figure 7 depicts sample AFB segmentation results. The R/G ratio and SVM methods gave by far the worst results overall. The ESFPNet-S model gave superior segmentation and precision performance results over all other models. In addition, the ESFPNet-S model’s 0.940 recall nearly matches the SSFormer-L model’s 0.949 recall. More specifically, for the AFB test set, the SSFormer-L and ESFPNet-S models detected 111 and 110 ground truth regions, respectively, over the 53-frame AFB test set, which contained 117 ground truth lesion legions. The seven regions missed by ESFPNet-S tended to be small (<1000 pixels) and/or appeared darker (less illuminated) and blurred, with the largest missed region made up of 10,383 pixels. Notably, ESFPNet-L exhibited slightly lower performance than ESFPNet-S. This could be attributed to (1) its significantly more complex Mit-B4 encoder, which was originally designed for the SegFormer to segment the much larger 1024 × 2048 cityscapes images, and (2) the correspondingly more complex ESFP decoder [24,46], i.e., the larger model implicitly requires more data to optimally train it. We also note that only the R/G ratio, SVM, and Unet++ methods detected any false positive regions on a normal frame.

Regarding the segmentations in Figure 7, the ESFPNet-S model gave the best performance, with gradual declines in performance observed for the other deep learning models. Lastly, the R/G ratio method missed a lesion on frame #1627 of case 21405-195, whereas the SVM method consistently produced over segmentations in all examples.

### 3.2. Colonoscopy

We next considered the ESFPNet performance for the problem of defining lesion (polyps) in colonoscopy video. The study’s aim was to demonstrate our proposed model’s robust performance and adaptability to a different endoscopy domain.

For the studies, we drew on five highly cited public video datasets that have been pivotal in the evaluation of polyp analysis methods [18]. These datasets include CVC-ClinicDB [39], Kvasir-SEG [47], ETIS-Larib [48], CVC-ColonDB [3], and CVC-T [49]. The number of total video frames in these datasets ranged from 60 to 1000, similar in size to our AFB dataset.

Three distinct experiments, which considered learning ability, generalizability, and polyp segmentation, were completed using the datasets. The experiments mimicked the procedures performed by Wang et al. and Lou et al. for their respective SSFormer and CaraNet architectures [19,20]. For all experiments, we used the mean Dice and mean IoU metrics. For the generalizability experiment, we also considered the structural measurement $S_{\alpha }$ [50], enhanced alignment metric $E_{\phi }^{max}$ [51], and the pixel-to-pixel mean absolute error (MAE) metric as considered by Lou et al. [19]. All metrics again were computed using the evaluation tool provided with PraNet [41].

Learning ability experiment: We trained, validated, and tested the three ESFPNet models, along with the Unet++, DeepLabv3+ [52], MSRF-Net, and SSFormer-L models. Each model was trained and validated with data from a particular database. Each model was then tested on a test subset from the same database. This gave an indication of the model’s learning ability to make predictions on previously seen data. We followed the experimental scheme used for the MSRF-Net [53]. In particular, using the CVC-ClinicDB (612 frames) and Kvasir-SEG (1000 frames) datasets, we randomly split each dataset into three subsets: 80% train, 10% validation, and 10% test. Following the same training procedures as for the AFB tests, we froze a model when it optimized the mean Dice measure on the validation dataset. The frozen models were then used to generate prediction results for the test dataset. For the models from other’s works, we used their reported results in the comparison. See Table 4. For the CVC-ClinicDB dataset, ESFPNet-S and ESFPNet-L gave the best and second best results, respectively, while, for the Kvasir-SEG dataset, ESFPNet-L and ESFPNet-S gave the second and third best measures, nearly equaling that of SSFormer-L. Overall, the experiment demonstrates the effective learning ability of ESFPNet.

Generalizability experiment: For the three proposed ESFPNet models, we conducted the following experiment. First, each model was trained on dataset #1. Next, each model was tested on dataset #2, data from a previously unseen source. In particular, we applied the same dataset splitting as recommended for the experimental set-up for the PraNet [41], i.e., 90% of the video frames constituting the CVC-ClinicDB and Kvasir-SEG datasets (1450 frames) were used for training. Next, all images from CVC-ColonDB (300 frames) and ETIS-LaribPolypDB (196 frames) were used for testing (the previously unseen datasets). We kept the best-attained performance for each dataset as a measure of a model’s forecasting performance on an unseen dataset.

Table 5 clearly shows the capability of ESFPNet for generalizability over all five metrics. The results demonstrate the proposed ESFP decoder’s sustained adaptability through the -T, -S, and -L models, as the MiT encoder increases in complexity from B0, B2, and B4. Notably, the ascending segmentation performance results illustrate that the proposed ESFP aligns well with the enhanced capabilities offered by the increased parameter count of the MiT encoder. Lastly, the results highlight our model’s capacity to assimilate common features of polyps from diverse datasets and predict effectively among unseen data.

Polyp Segmentation Efficacy: We used the same training dataset as in the generalizability experiment, where each model was separately trained until its loss converged. The remaining 10% of the video frames from the CVC-ClinicDB and Kvasir datasets (62 and 100 frames, respectively) and all images from CVC-T (60 frames), CVC-ColonDB (300 frames), and ETIS-LaribPolypDB (196 frames) were used for testing, giving five distinct test datasets. The focus of the experiment was to evaluate segmentation performance over both familiar and unseen data across five datasets. For the other models, we used the numerical results reported in the following studies: Unet++, Zhou et al. [22]; SFA, Fang et al. [45]; CaraNet, Lou et al. [19]; and SSFormer, Wang et al. [20]. Table 6 gives the results.

**Figure 8:**
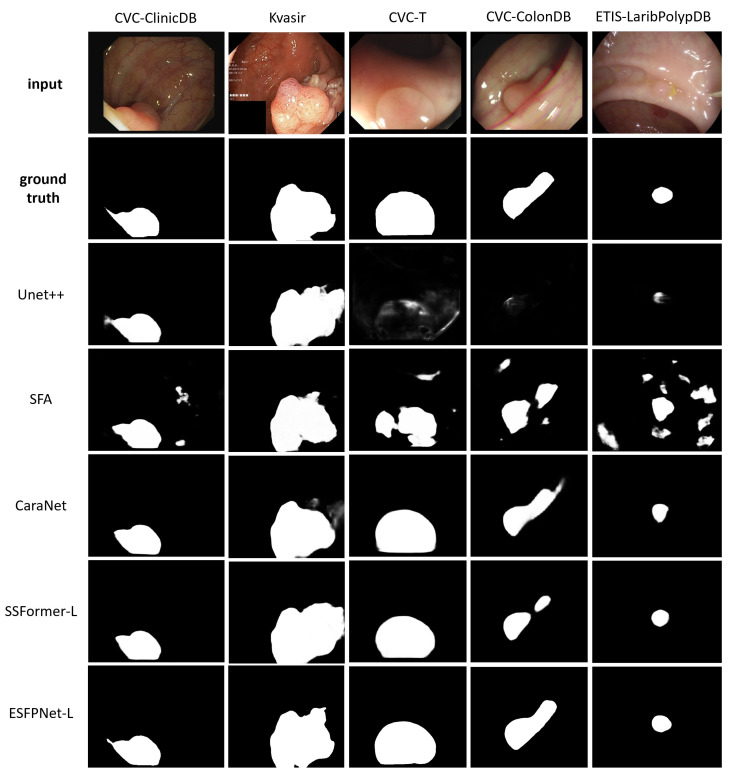
Polyp segmentation results for sample video frames taken from the following public polyp datasets: CVC-ClinicDB, Kvasir-SEG, CVC-T, CVC-ColonDB, and ETIS-LaribPolypDB. The first two rows depict the original video frames and ground truth segmentations. Rows 3 through 7 show segmentations derived by the various models, ordered with respect to their name in Table 6.

ESFPNet-L and ESFPNet-S gave superior performance for two unseen datasets (CVC-ColonDB, ETIS-LaribPolypDB) and one familiar dataset (Kvasir-SEG), respectively, with SSFormer-L giving the second best effort for two out of these datasets. The CaraNet gave the best performance results on the remaining two datasets (familiar CVC-ColonDB and unseen CVC-T), with ESFPNet-L and ESFPNet-S giving the second and third best performance results on these datasets. The sample lesion segmentations of Figure 8 anecdotally corroborate these numerical observations. The Unet++ and SFA models were not competitive in this test. Overall, the ESFPNet architecture gives exemplary segmentation performance over this diverse collection of datasets.

### 3.3. Computation Considerations and Ablation Study

The number of parameters defining a network gives a direct indication of the number of floating-point operations (FLOPs) required to process an input and, hence, its computational efficiency. Table 7 gives measures of model complexity and computational cost for seven of the network models studied in Section 3.1 and Section 3.2. The GFLOPs values were calculated using the fvcore.nn package under Facebook’s research platform [54]. With respect to the models which gave the best performance results in the previous tests, the ESFPNet-S model requires substantially fewer parameters and demands significantly less computation than CaraNet and SSFormer-L. Over all networks, the ESFPNet-T model requires by far the fewest number of parameters and processing operations. Since the earlier experiments indicate that ESFPNet-T can give potentially acceptable performance, its simplicity may warrant use in certain applications.

To gain a fuller picture of model practicality, we also considered the actual computation time in a real-world implementation. Because an end-to-end turnkey version of a network model requires additional image processing steps, such as cropping, resizing, and normalization (Section 2.1), the actual computation time depends on more than just a network’s parameter count. Secondly, the actual computation time is also influenced by the power of the CPU and GPU employed. Table 8 presents the computation time measurements for various CPU/GPU configurations, using the hardware discussed in Section 2.4.

Leveraging CPU multi-threading cuts 5–15 ms per frame by parallelizing the image preparation, resizing, and display operations, but overall, the computation time remains very high if the GPU is not used. GPU acceleration markedly decreases the overall computation time to a range of 26 to 88 ms per frame over all models. Lastly, adding CPU multi-threading to GPU processing cuts a substantial 10–15 additional ms per frame, giving a computation time range of 17 to 73 ms per frame—hence, CPU efficiency clearly helps significantly reduce the computation time and should not be neglected.

Table 8 shows that the superior performing ESFPNet-S model achieves a processing speed exceeding 30 frames per second, enabling real-time video processing, while ESFPNet-T achieves a processing speed of 48 frames per second. In addition, the ESFPNet-S exhibits the second-lowest parameter count and GFLOPs measure, per Table 7. While Unet++ exhibits the lowest parameter count, it demands the highest computational load of all models due to its dense convolution operations in skip-connections, which especially escalates with larger input sizes. Coupling this with its weaker analysis performance noted earlier, it is the least competitive of the network models. Notably, even though the ESFPNet-S and SSFormer-S models share the same backbone, ESFPNet-S requires fewer parameters and significantly fewer GFLOPs than the SSFormer-S while also giving better segmentation performance. Similar observations can be made when comparing the ESFPNet-L and SSFormer-L models. Although the CaraNet analysis performance is often comparable to that of ESFPNet-S, it demands more parameters and computational resources than ESFPNet-S.

To summarize, for the endoscopy applications considered here, the results and discussion of Section 3.1 and Section 3.2 clearly demonstrate the strong analysis performance of ESFPNet-S. In addition, as discussed above, the results of Table 7 and Table 8 show the architectural efficiency of ESFPNet-S, both in terms of the number of parameters required and computation time. Thus, ESFPNet-S strikes a favorable balance between analysis performance and architectural efficiency.

To conclude, we performed an ablation study of the ESFPNet-S model, which draws on the MiT-B2 encoder. In particular, we investigated the impact of each component comprising the model’s ESFP decoder (Figure 1). Table 9 gives the results (cf. Table 3). The table clearly shows that all components make a substantial contribution to the performance of the ESFP decoder.

## 4. Discussion and Concluding Remarks

Lung cancer, the world’s most common cause of cancer death, still tends to be detected at an advanced stage, resulting in a high patient mortality rate [55]. In addition, colorectal cancer continues to be the second largest cause of cancer death [7]. Hence, early-stage cancer detection is vital to increase patient survival. For both domains, endoscopy has proven to have considerable value as a minimally invasive tool for imaging precancerous and cancerous lesions along the walls of hollow tubular organs, such as the lung airways and intestinal tract. Unfortunately, the standard approach for performing an endoscopic exam demands human-based visual inspection of the resulting video to localize potential lesions—a very time-consuming, error-prone task, dependent on the widely varying skills of individual physicians.

For the airways, autofluorescence bronchoscopy (AFB) has the potential to be a superior tool for distinguishing potential cancerous lesion sites from normal regions. Yet, the aforementioned limitations of human-based inspection have largely limited the use of AFBs in academic centers, with AFB not regularly being used for lesion analysis [2]. While colonoscopy is a more common procedure, research toward finding faster and more robust automated methods, with the possibility of reducing the dependence on human skill, continues [18].

We have a proposed a deep learning model referred to as ESFPNet that enables efficient real-time analysis of endoscopic video for lesion detection and segmentation. When compared to existing methods for endoscopic video analysis, ESFPNet gave superior segmentation performance for an AFB video database. To the best of our knowledge, this is the first study to apply deep learning to AFB lesion analysis. (Our anonymized AFB dataset, the first of its kind, is available to the public under “Links/Public Databases” on our laboratory’s web site [38].) ESFPNet also gave superior segmentation performance results for three widely used public colonoscopy databases and comparable performance results to the CaraNet on two other public colonoscopy databases [19]. Notably, for the AFB dataset, ESFPNet also gave superior immunity to false positive lesion detections on normal frames, a common issue noted previously [34,35]. Because we prioritized lesion detection performance (unlike all previous deep learning colonoscopy studies), we included normal frames in the training, which helps attention-based networks, such as the ESFPNet, CaraNet, and SSFormer models, avoid false detections. In addition, further experiments with the publicly available colonoscopy datasets also indicated the ESFPNet model’s learning ability and generalizability.

Following on our earlier comments, a major challenge in the general field of endoscopic video analysis is the need to alleviate the demands and obvious accuracy concerns of human-based visual inspection during a live endoscopic procedure. To this point, devising real-time, or at least near real-time, methods for endoscopic video analysis remains a crucial goal, as such methods would allow the physician to immediately focus attention on the important video information contained in the vast oncoming video stream. This would then permit the physician to make more instantaneous and confident clinical decisions. Such methods would also facilitate more comprehensive endoscopic exams than those that are currently feasible based on visual inspection only—this would clearly further enhance the value and clinical success of endoscopic procedures. Overall, by addressing this challenge, such endoscopic procedures would become less skill dependent and burdensome, thereby enabling them to be performed more widely.

Our work has focused on the endoscopic video analysis task of real-time object detection and segmentation, where the objects specifically represent suspect cancerous lesions. However, current deep learning models demand substantial computational resources for accurate segmentation, making them often impractical for live use [56]. A major advantage of the ESFPNet is its computation efficiency. More specifically, its simpler lightweight model enables real-time usage and good segmentation performance as opposed to the CaraNet and SSFormer-L architectures, which are not suitable for real-time use and involve more complex models [19,20]. This property enabled the ESFPNet-S to process video frames at better than a real-time frame rate (30 frames/s) in our implementation.

The primary design innovation of the ESFPNet model lies in its novel efficient stage-wise feature pyramid (ESFP) decoder structure. In particular, the ESFPNet decoder begins with simple linear prediction through the basic prediction (BP) layer, which directly processes outputs from each encoder stage to generate useful features at various scales ($\frac{H}{4}$, $\frac{H}{8}$, …, $\frac{H}{32}$). Next through the aggregating fusion (AF) layer, the decoder utilizes a 1 × 1 kernel convolution layer to merge global and local features. A second linear prediction (AP) layer then follows, which guides local features at each scale to add more details to regions from global features at the corresponding scale. Finally, the multi-stage fusion (MF) layer accumulates region information, fully utilizing features at all scales, to produce the final output. Compared to the decoder structure of SSFormer, the ESFP decoder replaces the local emphasis (LE) layer with the BP layer to retain high-frequency feature information while reducing computation cost [20]; it also adds the MF layer to fully utilize features at every scale. In contrast to the SegFormer’s decoder structure, the ESFP decoder draws upon the AF and AP layers to provide more beneficial features that focus on flagging regions of interest (potential lesions) at all scales [24]; these features are then in turn utilized by the final MP layer.

Regarding future work, it would be helpful to add a lesion tracking mechanism that draws on the single-frame detection capability of ESFPNet to enable complete video sequence analysis, thereby more fully exploiting the information content of a complete sequence. As a related task, a method that automatically localizes the true 3D locations of identified lesions within the organ of interest would facilitate local treatment regimens and follow-up procedures. On a related note for lung cancer, researchers have noted that other bronchoscopic modalities, such as WLB and narrow-band imaging bronchoscopy, effectively complement AFB to facilitate potentially more robust multimodal detection of bronchial lesions [1]. We have been working toward the latter two tasks with the development of an early system prototype for multimodal bronchoscopic synchronization [57]. Lastly, along with computational performance, the interpretability of a network model’s output is important in helping physicians and researchers better understand and justify how a model identifies and segments important regions. To this point, the feature heatmaps we utilized for Figure 3 and Figure 4 illustrate the decision-making process of our model. This visualization technique was also used for SSFormer [20] and other related works. For live procedures, suitable visualization techniques and processing tools could be integrated into an interactive graphical system that gives live feedback on likely lesion locations. These techniques could include labeled bounding boxes on the live video, plots of region (lesion) segmentation metrics, and the selection of key frames featuring the “best” video view of a lesion, among others. We have made progress toward building an interactive system for AFB analysis that includes these techniques, but further work would be important for other applications [58].

Another important challenge in devising effective methods for endoscopic video analysis is the availability of suitably large datasets for training and validating candidate deep learning methods. While the datasets we used in our AFB and colonoscopy tests, both in-house and public, have similar sizes—and conform to the sizes of datasets used by many researchers in this field—they realistically are still insufficient for ascertaining a method’s performance in a high-volume live clinical setting. Unlike radiologic imaging scans, which do not require a complex operating room scenario for collection, endoscopic procedures arguably require a much greater dedication of clinical resources in terms of people, operating room preparation, and time to perform. In addition, after collecting such data, proper ground truth information must then be generated. Lastly, if such data are to be shared with the public and other researchers, they must be stripped of human identifiers to protect patient confidentiality. Ideally, as new high-volume clinical applications become mandated by physicians, such as the effective management of early lung cancer patients, larger multi-center clinical studies could help collect and manage such datasets. As a short-term alternative, because of the high cost of collecting live human video data, one could use semi-supervised learning methods, such as contrastive learning, to train candidate models more rigorously [59].

On a related comment, our model structure could be applied to other problems that draw on datasets of different dimensions from our tests. In particular, researchers could explore our network model for multi-class segmentation or multi-function tasks involving larger datasets or more complex cancer detection problems by leveraging the capabilities of MiT encoders and ESFP decoders. To this point, with respect to the MiT encoders, MiT-B4 employs more complex “transformer encoder” blocks (increasing number of encoder layers) in stages 2 and 3, as compared to the MiT-B2 encoder. In addition, both MiT-B2 and MiT-B4 use more intricate “transformer encoder” blocks than MiT-B0 at all stages. Therefore, these encoders give the capacity and flexibility to handle larger and more complex datasets [24]. Continuing, we note in turn that the ESFPNet efficient decoders are designed to match the actual configurations used for encoders. In fact, we demonstrated this design flexibility during our generalizability test in Section 3.2 (Table 5).

A final important challenge in the field of endoscopic analysis entails the ethical considerations involved in using deep learning (i.e., artificial intelligence [AI]) methods for making clinical decisions. As our focus here is on basic research in developing a new method that shows promise for accurate, efficient analysis, any future clinical deployment of our method (and others) as a clinic-ready “production mode” system certainly needs to address these issues. As our brief discussion of interpretability above highlighted, researchers have clearly recognized that caution is required in relying on the decisions made by so-called “black box” deep learning models. This issue had led to research in explainable artificial intelligence (XAI) [60,61,62]. This research has given rise to the imperative that complete systems deployed for clinical use should incorporate mechanisms that give interpretable models and explainable predictions. Such mechanisms are vital to ensure patient safety and decision-making transparency. Chaddad et al. summarize a number of these mechanisms, with the references giving detailed current surveys of this important area [60,61,62].

As an overall summary, our studies point to the combined superior analysis performance and architectural efficiency of the ESFPNet for endoscopic video analysis. We emphasize, however, that we have only tested our model on video from bronchoscopy and colonoscopy. Hence, we cannot categorically state that the model will give superior performance for other endoscopic video applications. For example, other endoscopic modalities, such as those drawing on hyperspectral imaging, have been explored for early cancer detection in the gastrointestinal tract, with some work having been undertaken toward applying deep learning to this imagery [63,64,65]. Yet, the learning ability and generalizability we demonstrated for ESFPNet in our results do give support to the belief that it could also be effective for application in other domains. 

## Figures and Tables

**Table 1:** Details for components constituting the ESFP decoder’s BP, AF, and AP layers per Figure 1. Figure 2 depicts module architectures. “Process” refers to the specific operations performed by each component.

BP Layer	BP #1	BP #2	BP #3	BP #4
Input	$F_{1}:\;\frac{H}{4}\times \frac{H}{4}\times C_{1}$	$F_{2}:\;\frac{H}{8}\times \frac{H}{8}\times C_{2}$	$F_{3}:\;\frac{H}{16}\times \frac{H}{16}\times C_{3}$	$F_{4}:\;\frac{H}{32}\times \frac{H}{32}\times C_{4}$
Process	**Linear_Layer**(·)	**Linear_Layer**(·)	**Linear_Layer**(·)	**Linear_Layer**(·)
Output	$F_{1}^{\mathrm{BP}}:\;\frac{H}{4}\times \frac{H}{4}\times C_{1}$	$F_{2}^{\mathrm{BP}}:\;\frac{H}{8}\times \frac{H}{8}\times C_{2}$	$F_{3}^{\mathrm{BP}}:\;\frac{H}{16}\times \frac{H}{16}\times C_{3}$	$F_{4}^{\mathrm{BP}}:\;\frac{H}{32}\times \frac{H}{32}\times C_{4}$
AF layer	AF #1	AF #2	AF #3	
Input	$F_{1}^{\mathrm{BP}}$ and $F_{2}^{\mathrm{AP}}$	$F_{2}^{\mathrm{BP}}$ and $F_{3}^{\mathrm{AP}}$	$F_{3}^{\mathrm{BP}}$ and $F_{4}^{\mathrm{BP}}$	
Process	1. Concat$\left(F_{1}^{\mathrm{BP}},U_{2}\left(F_{2}^{\mathrm{AP}}\right)\right)$	1. Concat$\left(F_{2}^{\mathrm{BP}},U_{2}\left(F_{3}^{\mathrm{AP}}\right)\right)$	1. Concat$\left(F_{3}^{\mathrm{BP}},U_{2}\left(F_{4}^{\mathrm{BP}}\right)\right)$	
	2. **ConvModule**(·)	2. **ConvModule**(·)	2. **ConvModule**(·)	
Output	$F_{1}^{\mathrm{AF}}=\frac{H}{4}\times \frac{H}{4}\times C_{1}$	$F_{2}^{\mathrm{AF}}=\frac{H}{8}\times \frac{H}{8}\times C_{2}$	$F_{3}^{\mathrm{AF}}=\frac{H}{16}\times \frac{H}{16}\times C_{3}$	
AP layer	AP #1	AP #2	AP #3	
Input	$F_{1}^{\mathrm{AF}}$	$F_{2}^{\mathrm{AF}}$	$F_{3}^{\mathrm{AF}}$	
Process	**Linear_Layer**(·)	**Linear_Layer**(·)	**Linear_Layer**(·)	
Output	$F_{1}^{\mathrm{AP}}=\frac{H}{4}\times \frac{H}{4}\times C_{1}$	$F_{2}^{\mathrm{AP}}=\frac{H}{8}\times \frac{H}{8}\times C_{2}$	$F_{3}^{\mathrm{AP}}=\frac{H}{16}\times \frac{H}{16}\times C_{3}$	

**Table 2:** AFB single-frame dataset, subdivided into train, validate, and test datasets. The complete dataset consists of 685 720×720 video frames. The column “Cases” indicates the number of patient airway exams used for a given data subset. The “Total frames” and “Split ratio” columns indicate the number of frames and the percentage of frames, respectively, that were allocated to a particular subset. For an entry of the form “A/B” in these two columns, “A” corresponds to lesion frames and “B” corresponds to normal frames. Lastly, the column “Size range” denotes the percentage of pixels within of a frame’s circular scan area that correspond to lesion regions.

Dataset	Cases	Total Frames	Split Ratio	Size Range
Train	10	97/223	47/47	0.3–54.3
Validate	5	58/139	28/29	0.2–75.1
Test	5	53/115	25/24	0.5–45.8

**Table 3:** AFB test results. Columns 2 and 3 measure segmentation performance, while Columns 4 and 5 give detection performance. The quantities “mDice” and “mIoU” refer to the mean Dice and mean IoU metrics, respectively. “SVM” refers to a support vector machine approach [12]. **Bold** numbers indicate the best measures.

Method	mDice	mIoU	Recall	Precision
R/G ratio	0.549	0.418	0.820	0.518
SVM	0.527	0.390	0.914	0.389
Unet++	0.722	0.587	0.897	0.653
CaraNet	0.745	0.610	0.855	0.858
SSFormer-S (B2)	0.746	0.612	0.923	0.778
SSFormer-L (B4)	0.737	0.604	**0.949**	0.799
ESFPNet-T (B0)	0.717	0.574	0.880	0.820
ESFPNet-S (B2)	**0.756**	**0.624**	0.940	**0.862**
ESFPNet-L (B4)	0.738	0.600	0.889	0.769

**Table 4:** Learning ability experiment. **Bold** values denote top performance.

	CVC-ClinicDB		Kvasir-SEG	
**Model**	**mDice**	**mIoU**	**mDice**	**mIoU**
Unet++	0.915	0.865	0.863	0.818
Deeplabv3+	0.888	0.871	0.897	0.858
MSRF-Net	0.942	0.904	0.922	0.891
SSFormer-L	0.945	0.899	**0.936**	**0.891**
ESFPNet-T	0.945	0.900	0.917	0.866
ESFPNet-S	**0.951**	**0.911**	0.929	0.884
ESFPNet-L	0.949	0.907	0.931	0.887

**Table 5:** Generalizability experiment with **bold** values marking the best outcomes.

Dataset	Model	mDice	mIoU	$S_{\alpha }$	$E_{\phi }^{\max}$	MAE
CVC-ColonDB	ESFPNet-T	0.781	0.699	0.843	0.895	0.036
	ESFPNet-S	0.795	0.711	0.854	0.905	0.032
	ESFPNet-L	**0.823**	**0.741**	**0.871**	**0.917**	**0.029**
ETIS-LaribPolypDB	ESFPNet-T	0.781	0.701	0.866	0.910	0.016
	ESFPNet-S	0.807	0.730	0.879	0.916	0.015
	ESFPNet-L	**0.827**	**0.752**	**0.892**	**0.935**	**0.011**

**Table 6:** Polyp segmentation prediction efficacy across five polyp datasets. **Bold** values indicate the best scores.

Model	CVC-ClinicDB		Kvasir-SEG		CVC-T		CVC-ColonDB		ETIS-LaribPolypDB	
	mDice	mIoU	mDice	mIoU	mDice	mIoU	mDice	mIoU	mDice	mIoU
Unet++	0.794	0.729	0.821	0.743	0.707	0.624	0.483	0.410	0.401	0.344
SFA	0.700	0.607	0.723	0.611	0.297	0.217	0.469	0.347	0.467	0.329
CaraNet	**0.936**	**0.887**	0.918	0.865	**0.903**	**0.838**	0.773	0.689	0.747	0.672
SSFormer-L	0.906	0.855	0.917	0.864	0.895	0.827	0.802	0.721	0.796	0.720
ESFPNet-T	0.912	0.859	0.905	0.802	0.884	0.817	0.775	0.695	0.755	0.677
ESFPNet-S	0.921	0.873	**0.921**	**0.874**	0.864	0.798	0.801	0.715	0.803	0.725
ESFPNet-L	0.928	0.883	0.917	0.866	0.902	0.836	**0.811**	**0.730**	**0.823**	**0.748**

**Table 7:** Model computational attributes. “Parameters” equals the number of model parameters in millions, while “GFLOPs” corresponds to gigaflops, which indicates the floating point operations required to process an input true-color video frame having dimensions 3 × 352 × 352 (3 RGB channels).

Model	Parameters	GFLOPs
Unet++	9.2	65.7
SSFormer-S (B2)	29.6	20.0
SSFormer-L (B4)	66.2	34.6
CaraNet	46.6	21.8
ESFPNet-T (B0)	3.5	1.4
ESFPNet-S (B2)	25.0	9.3
ESFPNet-L (B4)	61.7	23.9

**Table 8:** Computation time (milliseconds per frame) for various deep learning models under different hardware configurations. “CPU” implies using a single CPU, “CPU multi-thread” indicates performing multi-threaded processing, “CPU + GPU” denotes using the GPU with a single CPU thread, and “CPU multi-thread + GPU” represents using the full capability of the computer system discussed in Section 2.4.

	CPU Only	CPU Multi-Thread	CPU + GPU	CPU Multi-Thread + GPU
Unet++	632.1	624.1	26.7	17.0
CaraNet	521.3	506.7	76.1	66.8
SSFormer-S	441.8	436.6	44.4	32.0
ESFPNet-T	144.2	135.0	34.6	20.7
ESFPNet-S	369.1	360.1	44.4	31.9
ESFPNet-L	759.9	754.1	88.2	73.6

**Table 9:** Ablation study to evaluate the contribution of each decoder component of the ESFPNet-S model for AFB analysis. Per Figure 1, the ESFP decoders components are as follows: BP = basic prediction layer; AF = aggregating fusion layer; AP = aggregating prediction layer; MF = multi-stage fusion layer.

Decoder Components Used	mDice	mIoU
MF	0.707	0.567
BP + MF	0.732	0.596
BP + AF + MF	0.738	0.602
AF + AP + MF	0.720	0.583
ESFP (all used)	0.756	0.624

## Data Availability

The AFB dataset created for this study is publicly available under the “Links/Public Databases” link of our laboratory’s web site at www.mipl.ee.psu.edu.
